# Important aspects in relation to patients’ attendance at exercise-based cardiac rehabilitation – facilitators, barriers and physiotherapist’s role: a qualitative study

**DOI:** 10.1186/s12872-017-0512-7

**Published:** 2017-03-14

**Authors:** Maria Bäck, Birgitta Öberg, Barbro Krevers

**Affiliations:** 1 Department of Medical and Health Sciences, Division of Physiotherapy, Faculty of Medicine and Health Sciences, Linköping University, SE-581 83 Linköping, Sweden; 2 Department of Medical and Health Sciences, Division of Health Care Analysis, Faculty of Medicine and Health Sciences, Linköping University, SE-581 83 Linköping, Sweden

**Keywords:** Content analysis, Coronary artery disease, Exercise, Interviews, Patient perspective

## Abstract

**Background:**

In order to improve attendance at exercise-based cardiac rehabilitation (CR), a greater insight into patients’ perspectives is necessary. The aim of the study was to explore aspects that influence patients’ attendance at exercise-based CR after acute coronary artery disease (CAD) and the role of the physiotherapist in patients’ attendance at exercise-based CR.

**Methods:**

A total of 16 informants, (5 women; median age 64.5, range 47-79 years), diagnosed with CAD, were included in the study at the Cardiology Department, Linköping University Hospital, Sweden. Qualitative interviews were conducted and analysed according to inductive content analysis.

**Results:**

Four main categories were identified: (i) previous experience of exercise, (ii) needs in the acute phase, (iii) important prerequisites for attending exercise-based CR and (iv) future ambitions. The categories demonstrate that there are connections between the past, the present and the future, in terms of attitudes to facilitators, barriers and the use of strategies for managing exercise. An overall theme, defined as existential thoughts, had a major impact on the patients’ attitudes to attending exercise-based CR. The interaction and meetings with the physiotherapists in the acute phase were described as important factors for attending exercise-based CR. Moreover, informants could feel that the physiotherapists supported them in learning the right level of effort during exercise and reducing the fear of exercise.

**Conclusions:**

This study adds to previous knowledge of barriers and facilitators for exercise-based CR that patients with CAD get existential thoughts both related to exercise during the rehabilitation process and for future attitudes to exercise. This knowledge might necessitate greater attention to the physiotherapist-patient interaction. To be able to tailor exercise-based CR for patients, physiotherapists need to be aware of patients' past experiences of exercise and previous phases of the rehabilitation process as these are important for how patients’ perceive their need and ability of exercise.

**Electronic supplementary material:**

The online version of this article (doi:10.1186/s12872-017-0512-7) contains supplementary material, which is available to authorized users.

## Background

Coronary artery disease (CAD) remains the leading cause of morbidity and mortality worldwide [1]. As the number of fatalities after an acute coronary event has fallen, partly as a result of improved medical care and the reduction of CAD-related risk factors, patients in need of secondary prevention are growing in number [2]. Cardiac rehabilitation (CR) is an important part of secondary prevention for patients with CAD, including multifactorial interventions designed to optimise physical, psychological and social function, in addition to stabilising, slowing, or even reversing the progress of atherosclerotic disease [3, 4]. CR services are provided through an interdisciplinary approach and include specific core components such as baseline patient assessment, nutritional counselling, risk factor management, psychosocial interventions, physical activity counselling and exercise [3].

Exercise has been identified as a key factor in CR and physiotherapists therefore play an important role in prescribing individually tailored exercise programmes [5]. Studies have clearly demonstrated the impact of exercise-based CR in terms of marked reductions in cardiac mortality and hospitalisation, as well as favourable effects on cardiac risk markers and psychological factors [6, 7]. Despite well-known positive effects of exercise-based CR, attendance rates are notoriously low; less than 50% worldwide [8] and 52% in Sweden in 2016 [9].

In the light of the secondary prevention of CAD, it is important to note the theoretical distinction between exercise and physical activity. These constructs are often used interchangeably, although physical activity has been defined as “any bodily movement, produced by skeletal muscles, that results in energy expenditure”, while exercise has been defined as “a subset of physical activity that is planned, structured, repetitive and purposeful in the sense that the improvement or maintenance of physical fitness is the objective” [10]. Several factors, such as the initial level of physical fitness and the dose of exercise, i.e. frequency, duration and intensity, influence the outcomes of exercise [11].

Predictors of low attendance rates at multifactorial CR have mainly been studied through quantitative analysis and are usually categorised into medical factors, health-care system factors and patient-oriented factors [12–17]. Qualitative research can complement this knowledge, giving a greater insight into the patients’ perceptions of the phenomenon [18, 19]. Meta-synthesis of qualitative data in the context of multifactorial CR has shown that key factors for non-attendance could be categorised into individual factors and contextual factors.

There is, however, a lack of studies focusing primarily on patient perceptions of facilitators and barriers to attending exercise-based CR and the role of the physiotherapist in this context.

In order to improve exercise-based CR which matches the needs of patients, a deeper understanding of patient perspectives is necessary. The aim of the study was to explore aspects that influence patients’ attendance at exercise-based CR after acute CAD and the role of the physiotherapist in patients’ attendance at exercise-based CR.

## Methods

The reporting of data in this study follows the consolidated criteria for reporting qualitative research (COREQ) checklist [20].

### Design

This is a qualitative study with semi-structured interviews analysed according to inductive content analysis [21].

### Informants

Patients (informants) with a principal diagnosis of CAD were recruited at the cardiac intensive care unit (CCU) or at the exercise-based CR at Linköping University Hospital, Sweden, between autumn 2014 and spring 2015. To increase the variation in views, the patients were selected via a relevance sampling strategy [21] based on age, gender and various experiences of exercise after acute CAD, i.e. both attenders and non-attenders at exercise-based CR. Patients with coronary artery bypass grafting were not available at the CCU and therefore not included in the study. The exclusion criteria were an inability to speak or understand the Swedish language, serious physical or psychological diseases interfering with participation. The intention was to include 15-20 participants, as this was considered appropriate in order to capture both unique variations and common patterns within a study of this type and scope. A total of 16 patients, (5 women; median age 64.5, range 47-79 years) took part in the study. Four of the informants had diagnosis of ST-elevation myocardial infraction (STEMI), seven of the informants had diagnosis of non-STEMI and five informants had performed a percutaneous coronary intervention (PCI) due to instable angina pectoris (Table 1). The regional ethical review board in Linköping approved the study protocol and all the patients gave their informed consent prior to participation.

**Table 1:** Descriptive characteristics of the informants (*N* = 16)

Gender, women, n	5
Age, years, median (range)	64.1 (47-79)
Type of CAD, n	
STEMI	4
Non STEMI	7
Unstable angina	5
Attendance at exercise-based CR, n	9
Civil status, n	
Married/cohabitant	11
Living alone	5
Work, n	
Professional employment	7
Retired	7
Internal missing	2

*n* number, *CAD* coronary artery disease, *STEMI* ST elevation myocardial infarction, *PCI* percutaneous coronary intervention due to instable angina pectoris, *CR* cardiac rehabilitation

### Setting

This study focuses exclusively on exercise-based CR. At the cardiac intensive care unit, key components for the physiotherapist include risk factor identification, physical activity counselling and patient referral to an early-outpatient CR programme, including exercise and interdisciplinary patient education and information. The out-patient CR programme (phase II CR) starts as soon as possible after discharge from hospital. The exercise programme is individually prescribed by physiotherapists, based on tests of physical fitness, and includes aerobic exercise at least 20 minutes, at 50-80% of VO_2max_ (13-17 on Borgs rate of perceived exertion scale) and muscular exercise, 8-10 upper and lower body exercises performed in 1-3 sets of 10-15 repetitions, two to three times a week for three to six months, in line with international guidelines [22]. Late-outpatient management comprising additional tests of physical fitness is offered to patients after one year. In Sweden, most health care is publicly funded. For all outpatient care, the patient pays a maximum fee of 122 EUR (133 USD) for a twelve-month period.

### Procedure

Patients meeting the inclusion criterion were enrolled by physiotherapists working at the cardiology clinic at Linköping University Hospital. The first author then contacted the patients by phone and provided further information about the study. If the patients were interested in participating, additional written information was sent by post and an appointment for the interview was booked. Face-to-face, in-depth, semi-structured interviews were conducted in a secluded room at the cardiology department. An interview guide was developed with the emphasis on patients’ views of their ability to participate in and attitudes to exercise-based CR and their perceptions of the role of the physiotherapist in exercise-based CR. Please find the detailed interview guide as Additional file 1. The main topics supported the interview as overarching questions to start the dialogue. The follow-up questions were aimed to deepen the dialogue if necessary. The interview guide was tested in a pilot interview. No significant changes were made and the pilot interview was included in the analysis. The interviews lasted for a median of 45 minutes (range 32 to 73) and were recorded digitally and transcribed verbatim.

### Analysis

The interview data were analysed using qualitative content analysis, as described by Krippendorff [21], using an inductive and text-driven approach. This type of content analysis does not require an underlying theory and an inductive approach is recommended when there is not enough former knowledge about the phenomenon [21]. The first step in the analysis process included reading the interviews thoroughly several times to obtain a sense of the whole. The next step was to classify meaning units in the text by preserving their core intent and labelling them with a code. The codes were sorted and abstracted into categories and sub-categories. Finally, the underlying meaning in the categories was linked together to create a theme. The first author (MB) conducted the interviews. Two researchers (MB and BK) independently performed a preliminary analysis and the results were compared. If differences were found, they were discussed until consensus was reached. This was done in an iterative process during the analysis. The results of the study were also presented to and discussed in the same manner with the other co-author (BÖ). All authors were independent from working with exercise-based CR in Linköping and were not involved with the patients’ care and rehabilitation. The first author has experience of working with exercise-based CR in other settings and in research. BÖ is an experienced physiotherapist and researcher and BK is an occupational therapist and experienced researcher with competence in qualitative methods. None of BÖ and BK has previously worked in the field of cardiology.

## Results

**Fig. 1:**
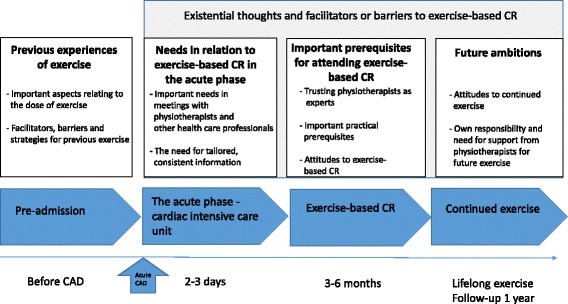
Figure of main categories, sub-categories and overall theme

The results are presented in five main categories, nine sub-categories and an overall theme, see Fig 1.

The categories demonstrate that there are connections between the past, the present and the future. These phases include different conditions and requirements that influence the patients’ ability or will to attend exercise-based CR. Physiotherapists were described as playing an evident role in some of these phases.

### Previous experience of exercise

The informants’ different previous experience of exercise influenced their attitudes to exercise during the exercise-based CR process.

#### Important aspects relating to the dose of exercise

Informants could describe a regular high dose of exercise as a natural part of their lives and said that it also remained as such throughout the exercise-based CR, even though the perceived importance of dose of exercise sometimes changed after the incidence of acute CAD.

*“It has always been obvious to me to exercise regularly to feel good. But now it is even more important after the myocardial infarction, to make sure it does not happen again” (2).*

Exercise was considered as a part of the occupation by informants with a physically demanding job and they did not believe that further exercise in their leisure time was necessary. Informants with no previous experience of exercise described it as boring and looked at themselves as “not the sporty type”. These informants considered daily physical activity, such as walking and gardening, as satisfying and good enough.

*“The physiotherapist asked me if I wanted to participate in exercise-based CR. I declined, because I do not think I have any need to exercise. I think I have always been sufficiently physically active anyway, with gardening” (9).*

#### Facilitators, barriers and strategies for previous exercise

The informants described previous experience of facilitators, barriers and strategies for taking part in exercise, which influenced their present situation.

Previous facilitators for exercise were described as support and motivation from relatives and friends, which remained important through the exercise-based CR process. Another previous facilitator for exercise was to set motivating goals, such as weight control or to increase physical fitness. These goals could be defined by the informants themselves but also in consultation with relatives and previous contact with health-care professionals. Moreover, the significance of choosing stimulating and fun exercises was highlighted.

*“I knew that exercise-based CR would suit me fine, as I had previously participated in similar exercise that I liked” (6).*

Previous barriers to exercise could be described as lack of time. Strategies discussed to overcome this barrier included the importance of proximity to exercise and to drawing up an exercise schedule for the week. Similar strategies were also described by the informants in the exercise-based CR process. Previous physical impairments created various barriers to exercise and the informants had different strategies for managing them, such as adapting the dose and type of exercise to their present physical function. Fear of exercise was expressed by informants who had experienced previous angina pectoris precipitated by exertion, in particular if they had friends with the onset of a myocardial infarction during exercise, or if they believed they ran an increased risk of heart disease due to genetic predisposition. High-intensity exercise was previously primarily avoided by these informants, fearing that something dangerous would happen to their hearts. Fear and avoidance of too strenuous exercise were also described by informants after the incidence of myocardial infarction, during exercise-based CR.

*“I was a little worried, because I have a twin brother who had a myocardial infarction eight years ago. After that, I stopped spinning, because I thought it wasn’t so good for the heart. During spinning, it is too easy to achieve a maximum performance” (6.)*

### Needs in relation to exercise-based CR in the acute phase

How well the physiotherapists and other health-care professionals met informants’ needs at the CCU influenced their decision to attend exercise-based CR.

#### Important needs in meetings with physiotherapists and other health-care professionals

Good interaction in meetings with physiotherapists was described by the informants as an important need when it came to attending exercise-based CR. Both the professional meeting and the content of the information on exercise were considered relevant. Receiving information both personally and in writing, together with an appointment time to exercise-based CR, were described as important factors for attending.

*“Being given an exact date and time for the exercise was great. I think that’s a good way to get people to participate” (6).*

The timing of the meeting with the physiotherapist was also described by the informants as an important aspect in relation to attendance at exercise-based CR. It was reported as easier to absorb information at certain times than others and that it was important that the physiotherapist was able to decide on the right time to convey information. Informants described the difficulty involved in absorbing a large amount of information by different health-care professionals especially when they perceived that health-care professionals were in a hurry, preventing them from asking questions or taking notes.

*“A lot of people in white coats arrived and told me things. And I had no paper to take notes” (2).*

The informants also described a need to receive support in order to manage psychological reactions that included thoughts of fear of dying and fear of movement. These reactions can influence the informants’ attitude to attending exercise-based CR. The informants saw the physiotherapists as people who were present and able to provide the right kind of support.

#### The need for tailored, consistent information

The informants described the importance of receiving consistent information from all the involved health-care professionals. If information on exercise-based CR provided by physiotherapists was underscored by other health-care professionals, this had a positive impact on the informants’ decision to attend. On the other hand, contradictory information about exercise given by different members of the health-care team was described as negatively affecting the informants’ decision to attend exercise-based CR. However, even though the informants did not understand all the information provided in the acute phase, they had complete trust in the competence of health-care professionals at the CCU at a university hospital.

Another factor described as important for attending exercise-based CR was that physiotherapists provided a tailored, strong recommendation that created an awareness of the potential future health benefits of participating in exercise-based CR. On the other hand, informants perceived that exercise-based CR was not important if the information was more vague and general.

*“I never understood why it was so important to attend exercise-based CR. Then I thought that I can do it myself instead. So I would have needed a clearer message which made the benefits of exercise evident and I recommend a carrot-and-stick policy to make patients with a myocardial infarction attend exercise-based CR. // Because I understand now that it is really important. So, it’s just stupid not to go there (11).*

### Important prerequisites for attending exercise-based CR

Important prerequisites for attending exercise-based CR, including the physiotherapist’s role, were described by the informants.

#### Trusting physiotherapists as experts

Physiotherapists could play a role as trusted experts in prescribing individualised exercise programmes and in providing adequate information about exercise. Informants could feel that the physiotherapists supported them in learning the right level of effort during exercise and reducing the fear of exercise.

*“It was reassuring that the physiotherapists were present. I thought I would never dare to run because my heart would start beating faster. It was definitely a threshold for me. But I got over that. I wasn’t afraid to run and sweat. It meant a lot to me” (12).*

The informants said that a sense of security was an important factor for attendance at exercise-based CR and that this could be partly mediated by the physiotherapists, as well as the context of performing exercise in a safe, controlled environment in hospital.

*“It feels secure in all cases to have trained physiotherapists who monitor and keep an eye on us, so to speak. It feels good to be here then. I can imagine that, if you start on other types of gymnastics, there will be a difference. Here you are in control” (1).*

Moreover, the motivation and support given by physiotherapists were described as an important part of exercise-based CR. Even though informants did not choose to attend exercise-based CR in hospital, they did welcome having their physical fitness tested by a physiotherapist to receive expert advice on continued exercise outside hospital.

*“It feels important to perform an exercise test. It can be a way to ensure that you do not actually fall over and die if you make an effort. So it is valuable, absolutely” (6).*

#### Important practical prerequisites

Factors that were described as facilitators for attending exercise-based CR for some informants could also be described as barriers by others.

Time was described as both a strong prerequisite and a barrier by the informants. The routine of having exercise schedules and the fact that attendance was controlled by the physiotherapists were described as facilitating factors, as the informants felt seen and observed. On the other hand, informants also found that time was a limiting factor, as the scheduled exercise programmes at hospital were not suitable for their overall life planning. Exercise programmes were seen to conflict with occupational demands and other social roles associated with housework and family life. The informants also said that it was unlucky to become ill just before the summer when there was a break in the normal exercise-based CR activities.

Proximity to hospital facilitated attendance, as it was possible to get to the hospital quickly and easily. While an opposite situation with long distances and difficulty with transportation were highlighted as barriers.

Financial costs were described as a barrier to attendance by informants who not yet had reached the maximum level for free health care in Sweden. On the other hand, other informants that had reached the maximum level described free health care as a facilitator for attending exercise-based CR.

An exercise group with a good size in which peers could show sympathy for one another was perceived by the informants as a significant prerequisite for attendance at exercise-based CR when it came to support and sharing experience.

*“You can put yourself in a context that there are more people who have suffered from these problems and some of them are perhaps even worse. You might not talk so much, but you feel that you are not the only one who has experienced this” (7).*

#### Attitudes to exercise-based CR

The informants’ self-image and self-efficacy were individual prerequisites that affected their attitudes to participate in exercise-based CR. Those with prior experience of exercise could express a high level of self-efficacy in terms of their ability to continue to exercise by themselves with no need of further physiotherapist guided CR.

Informants could express a strong will to feel normal and to live as normally as possible, which resulted in the decision not to attend exercise-based CR. Furthermore, informants said that, while attendance was seen to be appropriate for these other patients, it was not seen to be personally beneficial.

Age could be one of the aspects that influenced their attitudes to exercise-based CR. The informants felt that exercise in relation to heart disease was more important when they were younger, as compared to when they were getting older.

*“I participated in group exercise after my first myocardial infarction in 1996. But then I was younger, I was only 60 years old and it was no problem. But this time I said no. I don’t take exercise as seriously any more” (13).*

### Future ambitions

Informants described different attitudes and strategies for continued exercise, including how they perceived their own responsibility and how they needed support from the physiotherapist.

#### Attitudes to continued exercise

The informants expressed a more positive attitude to exercise after having finished exercise-based CR, which affected their future motivation to continue exercising. In addition, an awareness of other lifestyle risk factor modifications, such as a healthy diet, smoking cessation and reduced stress, was described to a greater extent by those with a positive attitude to exercise.

Another aspect that could influence the attitude to exercise was the desire to seize the moment. These informants described the importance of living for the day and they did not want to worry about tomorrow and, as a result, they did not regard future exercise or any other risk factor modifications as important.

Age could influence attitudes to continued exercise in various ways. One attitude to age was that the most important thing is to feel good when you get older, not to exercise. This was also supported by the thought that older people did not acquire any continuing health benefits from exercise. Another way to look at age was that keeping up with exercise was important for older people, because, if they become ill in the future, it is important to be in good condition.

*“Sometimes I say this to myself: ‘I‘m 77 years old and I have lived a good life and I do not think there is anything I need to change’. I think moderate physical activity, socialising with people and having a good time is important” (9).*

#### Own responsibility and need for support from physiotherapists for future exercise

The informants said that they themselves were primarily responsible for continued exercise after having finished exercise-based CR.

The intention to draw up a clear plan for continued exercise, including scheduled exercises alone, with friends or with relatives was highlighted.

Making exercise a habit was described as a strategy for increasing the potential for continued exercise after having finished exercise-based CR. Moreover, different kinds of goal setting was reported as important strategies for continued exercise, such as being able to resume previous exercise activities.

*“It has to become like a drug, about the same as using a seat belt is obvious. You have to miss it when you're not exercising, but you have to get there. It’s roughly the same as quitting smoking, it's not difficult at all. If you have decided it's no problem. But you have to want to do it” (15).*

Some perceived barriers to continued exercise relate to the informants’ worries about getting future physical impairments that will obstruct continuing exercise. The informants could be influenced by what they perceived as warning examples of future risk: seeing people who were physically inactive and what this might lead to, which increased their own motivation for exercise.

*“I see no obstacles to exercise. It could be your bodily function, but otherwise, no. It is only a question of deciding mentally” (6).*

*“I can sit at home and watch people walking by on the pavement and I think ‘No, I must never look like that 10 years from now.’ Yes, it's quite scary actually. So it’s good to watch people on the street. It motivates me, actually, it does. I become clearly motivated” (11).*

A desire for clearer support for continued exercise from the physiotherapist when finishing exercise-based CR was reported, such as how and where to continue exercise, or to be provided with a home-based exercise programme. Furthermore, the informants expressed a wish to be able to call the physiotherapists for further advice if necessary. The informants also said that it was motivating to be offered a long-term follow-up with the physiotherapist, to re-evaluate their physical fitness.

*“Testing physical fitness on one occasion doesn’t give so much. I would have preferred to perform the test on other occasions to see the results exercise can produce” (9).*

### Overall theme - Existential thoughts and facilitators or barriers to exercise-based CR

Existential thoughts had an impact on the patients’ attitudes to exercise throughout the rehabilitation process and also for the future. This theme was considered a basis for three of the other categories, including needs in the acute phase, important prerequisites for attending exercise-based CR and future ambitions.

After surviving the acute cardiac event, the informants said that they had been given another chance to live and that their priorities and perspectives for future choices in life had changed. Their increased awareness of mortality after the acute coronary event was described as a strong facilitator for attendance at exercise-based CR. Existential factors were also regarded as important motivating factors for the informants’ future attitudes to life-long exercise and other lifestyle risk factor modifications, to reduce the risk of another cardiac event. At the same time, existential factors could also be considered a barrier for exercise-based CR when informants ignored the future and instead described the desire to seize the moment and live for today. These informants did not want to worry about tomorrow or consider the importance of exercise.

Expressed existential thoughts were related to fear of exercise, in terms of fearing that something dangerous would happen to the heart, both in connection with previous experiences of exercise and during the rehabilitation process. These factors could influence the ability to attend exercise-based CR. In relation to fear of movement, physiotherapists could be described as trusted experts by providing psychological support, conveying a sense of security and prescribing individualised exercise programmes.

## Discussion

This study adds to previous knowledge that there are connections between the past, the present and the future in relation to barriers, facilitators and the use of strategies for managing exercise. Moreover, the results highlight the fact that the barriers described by some informants could also be described as facilitators by others. Physiotherapists were described as playing an evident role in some of the categories. Existential thoughts, defined as both motivators and barriers to exercise, had an impact on the patients’ attitudes to exercise throughout the rehabilitation process and also for exercise in future life.

It is known that cardiac events may be frightening and traumatic and that they could possibly become an existential threat that will trigger a chain reaction, resulting in the transformation of patients’ priorities and perspectives in life [23]. To the best of our knowledge, no previous study has reported on such a clear impact by patients’ existential thoughts when it comes to attending exercise-based CR and continued lifelong exercise. Existential thoughts were described as strong motivators related to patients’ beliefs that exercise could reduce the risk of another cardiac event and to patients’ strong desire to continue to live. A previous study has shown that a heightened awareness of mortality was a motivating factor that led patients with heart disease to make healthy lifestyle changes [24]. On the other hand, the current study also showed that existential thoughts could be regarded as barriers to attending exercise-based CR among patients who wanted to live in the present. These patients did not want to worry about the future and therefore did not regard participation in exercise-based CR as important. In relation to these findings, the role of patients’ existential dimension for participating in exercise-based CR must be further understood by physiotherapists and other health-care professionals.

Age was another factor that could be related to existential thoughts in the present study. Old age could be described in relation to a restricted future, for which patients wanted to create their own meaning. Some patients felt that exercise was meaningful in order to maintain or increase physical fitness as they age, while others believed they were too old to benefit from exercise. Older age has previously been associated with decreased attendance at exercise-based CR, mostly related to higher co-morbidity and transport difficulties [12, 15, 25]. Based on the results of the present study, it is, however, also important to understand the meaning of exercise for older patients with CAD from a broader perspective to increase the opportunity for a successful referral to exercise-based CR.

Furthermore, the patients described being confronted by existential thoughts related to fear of exercise, which encompasses patients’ fear of the life-threatening consequences of overstraining the heart. This fear of exercise was described in relation to both previous experiences of exercise before the cardiac event and in the acute phase following the cardiac event and during exercise-based CR. A previous study has shown that an excessive fear of movement, i.e. kinesiophobia, was present in 20% of patients with CAD and that kinesiophobia had a negative influence on variables important to rehabilitation outcomes [26]. Other studies have revealed that patients with CAD expressed concern about the risk of another myocardial infarction through physical exertion [23, 27]. To date, the design of a treatment intervention for kinesiophobia has not been studied extensively in patients with CAD. In patients with chronic pain, exposure *in vivo*, in which patients are required to perform the physical activities they fear the most with the goal of challenging catastrophic expectations, has been found to be an effective form of treatment [28]. The results of the present study showed that physiotherapists were described as trusted experts by providing psychological support, conveying a sense of security and prescribing individualised exercise programmes. Moreover, patients said that supervised tests of physical fitness, followed by an individually tailored exercise programme performed at hospital, reduced their fear of exercise. Extending this knowledge still further in quantitative studies is definitely an area for further research to help us address the clinical management of kinesiophobia in the context of exercise-based CR, in order to increase attendance at exercise-based CR.

With an understanding of the impact of the existential thoughts of patients with acute CAD in relation to exercise, the interaction between patients and physiotherapists becomes most important. This interaction has not previously been studied in the context of exercise-based CR. There are, however, previous studies defining factors with a key influence on the patient-physiotherapist interaction in musculoskeletal settings, showing that the physiotherapist’s communication and interpersonal skills, such as active listening, showing empathy and giving motivation and encouragement, were found to be crucial [29]. These factors also appear to be important in the patient-physiotherapist interaction in the present study as well as for the physiotherapist to decide on the right time to convey information. In addition, however, the present study highlights the fact that a meaning-centred existential perspective, starting from the patients’ own perceptions and experiences of exercise in relation to their heart disease, will further enrich the understanding of physiotherapy within the context of exercise-based CR. Building trust appears to be central in the patient-physiotherapist interaction. To succeed, we believe it is important to understand the aspects to which patients ascribe meaning in their meetings with physiotherapists and to use this information to deliver individualised care. This study showed that information on exercise-based CR provided by physiotherapists that was underscored by other health-care professionals had according to the informants a positive impact on their decision to attend. This is in line with results from previous studies, highlighting that the strength of a recommendation for exercise-based CR from a physician is a well-known factor to increase attendance [15].

In addition to previous knowledge, the results of the present study highlight the fact that the barriers described by some patients could also be described as facilitators by others. For example, the context of performing exercise in a safe, controlled environment in hospital, together with the positive social experience of peers, were highlighted as central prerequisites by some informants, while others found that current exercise-based CR programmes are rigid and difficult to attend.

Barriers to attendance at exercise-based CR in the present study were described in terms of time, distance and transport issues, co-existing medical problems, financial costs and age, which, in overall terms, match previous studies in the context of multifactorial CR [12–19, 30]. To enhance the access, adherence, target achievements and effectiveness of exercise-based CR programmes, there is a need for new initiatives targeting the needs of a broader range of patients, representative of usual clinical practice. For example, home-based programmes and eHealth solutions have the advantage of overcoming barriers of time and distance, but to date it is unclear whether these programmes reduce cardiovascular mortality to an extent similar to that of traditional hospital-based exercise-based CR programmes [6, 31, 32]. So, before implementation, the effects must be further studied in well-designed randomised controlled trials.

One strength of the current study with regard to trustworthiness is that the informants represent different genders, ages and experience of exercise, which contributes to a rich variation in the studied phenomena. To increase credibility, triangulation was used during the analysis, seeking agreement among co-researchers and, in addition, representative quotations from the transcribed text are presented. To confirm authenticity, the informants received information that the interviewer had no relationship to the present exercise-based CR clinic. Furthermore, the informants were not told that the interviewer had experience of clinical practice within exercise-based CR. Even though the findings of this study are linked to the present context, we believe they could be relevant and transferable to similar exercise-based CR settings.

## Conclusions

In conclusion, the findings of this study spotlight that existential thoughts, defined as both motivators and barriers to exercise, had an impact on the patients’ attitudes to exercise throughout the rehabilitation process and also for future exercise. There are connections between the past, the present and the future in relation to barriers, facilitators and the use of strategies for managing exercise. This knowledge can be used to make physiotherapists more aware of the impact of a patient-centred holistic approach in exercise-based CR. To be able to tailor exercise-based CR for patients, physiotherapists need to be aware of the connections between patients' past experiences of exercise and previous phases of the rehabilitation process as these are important for how patients' perceive their need and ability of exercise.

The results of this study can be translated into a quantitative design to develop and evaluate new treatment perspectives in exercise-based CR, with the overall aim of improving care and increasing attendance at exercise-based CR.

## Additional file

Additional file 1: Interview guide. Contains the detailed interview guide used in this study. (DOCX 20 kb)

## Abbreviations

CAD: Coronary artery disease
CR: Cardiac rehabilitation
