# Supplementary material for: Important aspects in relation to patients’ attendance at exercise-based cardiac rehabilitation – facilitators, barriers and physiotherapist’s role: a qualitative study
Source: BMC Cardiovasc Disord. 2017 Mar 14;17:77. doi: 10.1186/s12872-017-0512-7 (PMC5348799; doi:10.1186/s12872-017-0512-7)
Supplement: Additional file 1: — Interview guide. Contains the detailed interview guide used in this study. (DOCX 20 kb) [file 12872_2017_512_MOESM1_ESM.docx]

**Interview guide**

| **Main topics** | **Example of follow-up questions** |
| --- | --- |
|  |  |
| Informants’ perceptions about effects of exercise | - What did the physiotherapist at the cardiac intensive care unit tell you about exercise and exercise-based cardiac rehabilitation? - What is your view on taking part in exercise-based cardiac rehabilitation? - What is your perceptions about the impact of participating in exercise-based cardiac rehabilitation? - What is your view on how exercise affects health? |
| Informants´ expectations of support and own responsibility in relation to exercise. | - How do you view the need to exercise under the guidance of a physiotherapist after an acute coronary event? - How do you view the need to exercise on your own after an acute coronary event? - What do you think about the need for lifelong exercise? Can you describe potential barriers, facilitators and strategies? |
| Perceptions about the future | - Has the heart disease affected your view of the future? If so, how? - What is your thoughts about the importance of physical activity and exercise considering your view of the future? |
